# Evaluation of recently validated non-invasive formula using basic lung functions as new screening tool for pulmonary hypertension in idiopathic pulmonary fibrosis patients

**DOI:** 10.4103/1817-1737.56013

**Published:** 2009

**Authors:** Maha K. Ghanem, Hoda A. Makhlouf, Gamal R. Agmy, Hisham M. K. Imam, Doaa A. Fouad

**Affiliations:** *Department of Chest Disease, Faculty of Medicine, Assiut University Hospital, Assiut - 71111, Egypt*; 1 *Department of Diagnostic Radiology, Faculty of Medicine, Assiut University Hospital, Assiut - 71111, Egypt*; 2 *Department of Cardiology, Faculty of Medicine, Assiut University Hospital, Assiut - 71111, Egypt*

**Keywords:** Idiopathic pulmonary fibrosis, pulmonary hypertension, pulmonary function tests, screening

## Abstract

**BACKGROUND:**

A prediction formula for mean pulmonary artery pressure (MPAP) using standard lung function measurement has been recently validated to screen for pulmonary hypertension (PH) in idiopathic pulmonary fibrosis (IPF) patients.

**OBJECTIVE:**

To test the usefulness of this formula as a new non invasive screening tool for PH in IPF patients. Also, to study its correlation with patients' clinical data, pulmonary function tests, arterial blood gases (ABGs) and other commonly used screening methods for PH including electrocardiogram (ECG), chest X ray (CXR), trans-thoracic echocardiography (TTE) and computerized tomography pulmonary angiography (CTPA).

**MATERIALS AND METHODS:**

Cross-sectional study of 37 IPF patients from tertiary hospital. The accuracy of MPAP estimation was assessed by examining the correlation between the predicted MPAP using the formula and PH diagnosed by other screening tools and patients' clinical signs of PH.

**RESULTS:**

There was no statistically significant difference in the prediction of PH using cut off point of 21 or 25 mm Hg (*P* = 0.24). The formula-predicted MPAP greater than 25 mm Hg strongly correlated in the expected direction with O_2_ saturation (r = −0.95, *P* < 0.000), partial arterial O_2_ tension (r = −0.71, *P* < 0.000), right ventricular systolic pressure measured by TTE (r = 0.6, *P* < 0.000) and hilar width on CXR (r = 0.31, *P* = 0.03). Chest symptoms, ECG and CTPA signs of PH poorly correlated with the same formula (*P* > 0.05).

**CONCLUSIONS:**

The prediction formula for MPAP using standard lung function measurements is a simple non invasive tool that can be used as TTE to screen for PH in IPF patients and select those who need right heart catheterization.

Pulmonary hypertension is defined by a mean pulmonary arterial pressure over 25 mmHg at rest or over 30 mmHg during activity with accompanying increase of pulmonary vascular resistance over three Wood's unit.[1] It frequently complicates advanced IPF and is associated with poor outcome.[2–5] Currently, right-heart catheterization (RHC) is the gold standard test for the diagnosis of PH in patients with IPF. However, RHC is invasive and expensive.[2] Although echocardiography and CT-determined main pulmonary artery diameter are commonly used tests to screen for PH in patients with IPF, they are not always reliable.[3,6] Reliable, non invasive approaches to the diagnosis of PH in patients with IPF would improve patient safety, reduce costs and enable appropriate timing of RHC.[3]

Zisman *et al*. recently validated a new formula as a simple screening method for PH in IPF patients.[7] They combined the ratio of the forced vital capacity (FVC) percentage of predicted to diffusing capacity of the lung for carbon monoxide (DL_CO_) percentage of predicted and room air resting pulse oximetry (SpO_2_) in a linear regression formula to screen for PH in patients with IPF.[3] They had shown that a cut off of 25 mm Hg for the formula-estimated mean pulmonary artery pressure (MPAP) had sensitivity, specificity, positive predictive value (PPV) and negative predictive value (NPV) for PH (defined as mean pulmonary artery pressure [MPAP] from RHC greater than 25 mm Hg) of 71, 81, 71, and 81%, respectively. By selecting a lower cut off of 21 mm Hg for the formula-estimated MPAP, they maximized sensitivity (100%) for PH (defined as MPAP from RHC greater than 25 mm Hg) with the least compromise in specificity (40%). The researchers noted that clinicians could, depending on circumstances, select either the 21 mm Hg or the 25 mm Hg value as the threshold for confirmatory RHC testing.

Earlier, Steen *et al*.[8] followed a similar approach to that of Zisman and found that DL_co_ less than 55% pred and a ratio of FVC % pred/DL_co_ % pred greater than 1.4 were associated with PH; but, only 22% of patients fulfilling this criteria developed PH, in contrast to only two per cent without these criteria who developed PH. As internal validation does not guarantee adequate performance in other populations;[9] Zisman *et al*. further validated the PH screening formula in an external population of IPF patients and they along with other investigators recommended further testing of this formula to avoid selection bias from studying only those with more advanced illness.[7,10]

Hence, we found that studying these pulmonary function parameters, stressing on those included in the recently validated formula, might further clarify their possible role in predicting PH. To the current time pulmonary function tests (PFTs), despite being a non invasive tool for assessment of patients with different lung diseases, do not specifically contribute much to the assessment of PH. The aim of this study was to evaluate the new pulmonary functions-based formula as a new screening tool for PH in IPF patients with variable range of chest symptoms to study its possible usefulness as a simple non invasive screening tool.

Also, we wanted to study the correlation between the formula and patients' clinical characteristics, PFTs, ABGs and other commonly used screening tools for PH including ECG, CXR, TTE and chest CT angiography predictor measures to know if this formula could be a bedside tool in the outpatient and inpatient settings that enable clinicians' screening and follow-up of their patients for PH. This would probably avoid unnecessary costs of repeating other screening tests and possible risk of RHC.

## Materials and Methods

### Study setting, population and operational design

Thirty seven IPF patients were enrolled from a tertiary hospital in this prospective cross-section observation study. The IPF was diagnosed on the basis of clinical data, plain chest radiography, data from the high resolution CT scans (HRCT) of the chest and presence of restrictive pulmonary dysfunction.

This study was carried out through March 2008- February 2009. Patients were personally interviewed, given a 10-minute briefing on the aims of the study and then data collection was carried-out using a structured questionnaire and personal interview. The *first part* included personal data of patients, their characteristics and smoking history. The *second part* was about history of previous respiratory illnesses and current symptoms, the degree of dyspnea measured with the modified Medical Research Council (MMRC) dyspnea scale.[11] The *third part* was for clinical examination including symptoms and signs of PH, and investigations including CXR, ABGs, PFTs, ECG, TTE, HRCT and CTPA.

### Pulmonary function testing and ABG analysis

Measurement of spirometric data was taken as the best from at least three satisfactory spirometric tracings by the same technician using the same spirometer. Spirometry was performed using Zan 300 USB body plethysmography (Oberthulba, Germany). Standard methods for test performance and interpretation were used.[12,13] Forced vital capacity (FVC), forced expiratory volume in first second (FEV_1_), forced expiratory flow (FEF_25-75%_) and FEV_1_/FVC were measured. Lung volumes and diffusion tests were also recorded. The results were then expressed as percentage of predicated normal values for each subject after adjustment for age, sex and height.

Arterial blood gases on room air were obtained by blood sample from radial artery and analyzed using automated blood gas analyzer (Rapid Lab 855, Chiron diagnostics; Medfield, MA).

### ECG

We assessed electrocardiogram criteria for predicting PH as previously agreed[11,12,14] including;

- right-axis deviation;
- a tall R wave and small S wave in lead V1;
- rSR' pattern in lead V1 and a large S wave and small R wave in lead V5 or V6;
- ST-T segment depression and/or inversion are often present in the right precordial leads;
- right atrial enlargement is manifested as a tall P wave (≥2.5 mm) in leads II, III and aVF.

### Trans thoracic echocardiography

Resting TTE was performed and interpreted in all 37 patients using standard techniques by a specialized cardiologist. The trans-tricuspid pressure gradient was calculated using the modified Bernoulli equation (4ν^2^) where ν is the maximum velocity of the tricuspid valve regurgitant jet. Right atrial pressure (RAP) was estimated by respiratory variation in the diameter of the inferior vena cava and was categorized as 5, 10 or 15 mmHg. Right ventricular systolic pressure (RVSP) was calculated by adding the transtricuspid pressure gradient to the RAP estimate.

Direct and indirect signs of pulmonary hypertension[14–18] were looked for: Pulmonary blood flow velocity, paradoxical septal motion (septal bowling or flattering), pericardial effusion, right ventricular hypertrophy, RVSP and reduced right ventricular ejection time.

According to Arcasoy *et al*.,[17] a “positive” TTE for PH was defined as RVSP estimate greater than 45 mm Hg, the presence of RV dilation, dysfunction or hypertrophy.

### Radiological evaluation

Radiological assessment of radiological predictor measures of PH in plain CXR and CT chest were interpreted and scored by a specialized radiologist who was unaware of the patient's clinical and investigation data.

#### Chest X ray

Routine CXRs were done for the 37 IPF patients.

The following predictors for PH were assessed:

- Right descending pulmonary artery (RDPA) width greater than 16 mm, and the left descending pulmonary artery (LDPA) width diameter greater than 18 mm.[19,20]
- Hilar width greater than 10.5 cm.[21]
- Hilar/thoracic index greater than or equal to 35.[22]
- Cardiothoracic ratio greater than 43.[22]

#### High resolution CT chest examination

CT scans were obtained with the CT unit (PICKER PQ 2000S or TOSHIBA xpress/ SX). First, conventional high resolution CT chest examination was performed with 10mm thick sections obtained at 10mm intervals from the lung apex to the diaphragm (1-second scanning time, 130kv, 300mA). All images were viewed at lung (window width, 1600 HU; window level, −600 HU) and mediastinal (window width, 900 HU; window level, 100 HU) window settings.

#### Spiral CT pulmonary angiography

After HCCT examination, a contrast-enhanced evaluation of the pulmonary arteries with a spiral CT technique was done. The pulmonary angiogram protocol consisted of intravenous injection of 80-100 ml non-ionic contrast media at a flow rate of three ml/sec using a power injector. Slice width was three mm, increment size 1.5, and pitch 0.9.

Assessment: The CT predictors of PH as described in earlier studies were evaluated and scored as follows:[23–25] Main pulmonary artery diameter [MPAD] was measured at its widest dimension on the supine full-chest sequence. At this same level, the widest aorta diameter (AD) was measured and the MPAD/AD ratio was calculated. Diameters of the right and left main branches of the pulmonary artery, arterio-bronchial ratio (A/B ratio) (ratio between the bronchi and their accompanying arteries) were also measured. The position of the inter-ventricular septum was estimated on the CT angiography axial and reconstructed images and graded as normal (deviated to the right ventricle), straight or deviated to the left ventricle. The HRCT findings were summarized as absent or present.

CT measures for PH were considered positive if one or more of the following measures were present:[26]

- MPAD diameter greater than 29 mm with tapering of peripheral pulmonary arteries.
- MPAD/AD greater than 1
- A/B ratio greater than 1
- Straight or deviated inter-ventricular septum.
- Presence of bronchial collaterals

### Calculation of pulmonary pressure using formula

According to Zisman *et al*. 2008,[7] the following equation was used to calculate the predicted MPA P (in millimeters of mercury):

MPAP = −11.9 + 0.272 × SpO_2_ + 0.0659 × (100 − SpO_2_)_2_ + 3.06 × (percentage of predicted FVC/percentage of predicted DL_co_).

#### Ethical considerations

The Medical Ethics Committee of the hospital approved the protocol and a written consent to be enrolled in this study and to undergo scheduled investigations including CTPA, TTE, was obtained from all the patients or their next of kin.

### Statistical analysis

Numerical values are presented as mean plus/minus (SD) unless otherwise stated. Chi square or the Fisher's exact test, if cell sizes are small, was used in the 2 × 2 data. We compared mean values of all putative predictors of PH in the studied patients (formula-predicted positive and negative for PH) using the Student t test. We also studied correlation between the formula-predicted MPAP using two cut off points; 21 and 25 mmHg, and each of the putative predictors of PH (patients' clinical data, PFTs parameters, resting ABGs on room air and the ECG, CXR, TTE data and chest CT predictors of PH).

After regression of MPAP (obtained from 25 cut off point predicting formula) as a continuous variable on other or alternate predictors, in a multivariable linear regression model, MPAP prediction ability of the formula was assessed by model R2 in each case. Specifically, we examined the impact of adding the following variables to the model: Radiographic scores, PFTs variables, ABGs and TTE-measured RVSP.

All tests were two-tailed unless otherwise stated, and *P* values less than 0.05 were required for statistical significance. All statistical analyses were performed using statistical software (SPSS version 11) and the on line Epi-calc 2000 for test of proportions calculations (z test).

## Results

### Patient characteristics

The baseline characteristics of the study population (n = 37) are presented in Table 1. The patients tended to be females with an average age of 44.62 ± 16.57 years. Majority of the patients had chest symptoms - mostly cough and dyspnea. Cor pulmonale and central cyanosis were present in 67.6% and 89.2% of the study sample respectively, and only 27% of the study sample had clinical signs of pulmonary hypertension. Also, PFTs as well as ABGs parameters showed reduced values compared to expected predicted and normal values. Nearly half of the patients (45.9%) had normal ECG tracings. Mean TEE RVSP was 43.44 ± 17.84 and the mean equation calculated MPAP was 34.59 ± 21.35.

**Table 1:** Descriptive statistics for major characteristics*

Characteristics	Study sample (n=37) n (%)
Age (yr), mean (SD)	44.62 (16.57)
Sex (M/F)	7/30 (18.9/81.1)
Smoking	
Non smokers	31 (83.8)
Ex-smokers	6 (16.2)
Chest symptoms	
Cough	34 (91.9)
Grade 1 dyspnea	1 (2.7)
Grade 2 dyspnea	1 (2.7)
Grade 3 dyspnea	25 (67.6)
Grade 4 dyspnea	10 (27)
Corpulmonale	25 (67.6)
Central cynosis	33 (89.2)
Clinical signs of PH	10 (27)
Electrocardiogram findings	
Normal	17 (45.9)
P-pulmonale	17 (45.9)
Rt ventricular strain	3 (8.1)
Pulmonary function tests, mean (SD)	
FEV_1_, L	1.11 (0.4)
FEV_1_, % predicted	49.11 (15.44)
FVC, L	1.46 (0.55)
FVC, % predicted	53.11 (18.42)
FEV_1_/FVC	91.59 (16.34)
TLC, L	3.24 (1.15)
TLC, % predicted	72 (22.08)
DL_CO_, mL/mm Hg/min	4.06 (2.07)
DL_CO_, % predicted	57.38 (31.28)
Room air resting arterial blood gases, mean (SD)	
O_2_ saturation	85.88 (9.82)
O_2_ tension	55.07 (17.26)
CO_2_ tension	39.15 (7.8)
Formula-predicted MPAP, mean (SD)	34.59 (21.35)
Echo measured RVSP, mm Hg, mean (SD)	43.44 (17.84)

* Data are presented as mean (SD) or %. Patients characteristics were available in all of studied 37 patients; PH = pulmonary hypertension, FEV_1_ = forced expiratory volume in first second, FVC = forced vital capacity, TLC = total lung capacity, DL_CO_ = diffusing capacity of the lung for carbon monoxide, RVSP = right ventricular systolic pressure as measured by trans thoracic echocardiography,

### Patient characteristics based on formula-predicted presence of PH using 25 mmHg versus 21 mmHg cut off points

Table 2 compares patients' characteristics using two cut off points, 25 and 21 mmHg, where no statistically significant difference between the mean values and percentages of different parameters was found (*P* > 0.05).

**Table 2:** Patient characteristics based on formula-predicted presence of pulmonary hypertension using 25 mmHg versus 21 mmHg cut off points#

Characteristics	Formula predicted PH using (higher cut off point) 25 mmHg n (%)	Formula predicted PH using (lower cut off point) 21 mmHg n (%)	*P* value
No patients predicted to have PH			
Age (yr), mean (SD)	19	25	0.24
	44.79 (16.15)	47.68 (16.89)	0.57
Sex (M/F)	4/15	6/19	0.89
Smoking			
Non smokers	16 (84.2)	20 (80)	0.97
Ex-smokers	3 (15.8)	5 (20)	0.97
Chest symptoms			
Cough	19 (100)	25 (100)	
Grade 1 dyspnea	1 (5.3)	1 (4)	0.6
Grade 2 dyspnea	0 (0)	0 (0)	
Grade 3 dyspnea	12 (63.2)	16 (64)	0.8
Grade 4 dyspnea	6 (31.6)	8 (32)	0.77
Corpulmonale	14 (73.7)	18 (72)	0.83
Central cynosis	19 (100)	25 (100)	
Clinical signs of PH	6 (31.6)	8 (32)	0.76
Electrocardiogram findings			
Normal	8 (42.1)	10 (40)	0.87
P-pulmonale	11 (57.9)	15 (60)	0.87
Right ventricular strain	2 (10.5)	2 (8)	0.81
Pulmonary function tests, mean(SD)			
FEV_1_, L	1.00 (0.4)	1.05 (0.42)	0.69
FEV_1_, % predicted	46.16 (14.48)	48.16 (15.24)	0.66
FVC, L	1.37 (0.56)	1.41 (0.58)	0.29
FVC, % predicted	51.58 (17.14)	53 (17.83)	0.82
FEV_1_/FVC	87.16 (20.18)	89.56 (18.32)	0.79
FVC% pred/DL_CO_% pred	1.56 (0.91)	1.43 (0.85)	0.63
TLC, L	3.17 (1.39)	3.1 (1.24)	0.86
TLC, % predicted	71.21 (23.00)	69.12 (21.1)	0.76
DL_CO_, mL/mm Hg/min	3.23 (1.7)	3.51 (1.78)	0.6
DL_CO_, % predicted	44.58 (24.7)	49.24 (26.8)	0.55
Room air resting arterial blood gases, mean (SD)			
O_2_ saturation	78.72 (8.38)	81.22 (8.59)	0.34
O_2_ tension	43.6 (10.12)	45.9 (10.01)	0.46
CO_2_ tension	39.46 (10.39)	39.27 (9.12)	0.95
Formula-predicted MPAP, mean (SD)	48.66 (21.7)	42.58 (21.86)	0.37
Echo measured RVSP, mm Hg, mean (SD)	52.38 (19.68)	48.62 (18.54)	0.53

# Data presented as mean (SD) or %. Patients characteristics were available in all of studied 37 patients; PH = pulmonary hypertension, FEV_1_ = forced expiratory volume in first second, FVC = forced vital capacity, TLC = total lung capacity, DL_CO_ = diffusing capacity of the lung for carbon monoxide, RVSP = right ventricular systolic pressure as measured by trans thoracic echocardiography

### Comparisons of patients with and without PH based on the formula-predicted presence or absence of PH using 25 mmHg cut off point

Formula-predicted patients with and without PH did not differ with respect to age, gender, smoking history, chest symptoms and clinical signs of cor pulmonale or PH [Table 3]. As expected, those with formula-predicted PH had significantly lower DL_co_ [Figure 1a] and resting room air oxygen saturation SpO_2_ [Figure 1b] and partial arterial oxygen tension (PaO_2_) tension and significantly higher FVC% pred/ DL_CO_% pred [Figure 1c] and formula-predicted MPAP and TTE measured RVSP [Figure 1d] than those without PH [Figure 1]. However, they did not perform significantly worse on the rest of spirometry data. CXR and CT-derived scores suggestive of PH did not differ significantly between those with or without formula-predicted PH [Table 3].

**Figure 1a:**
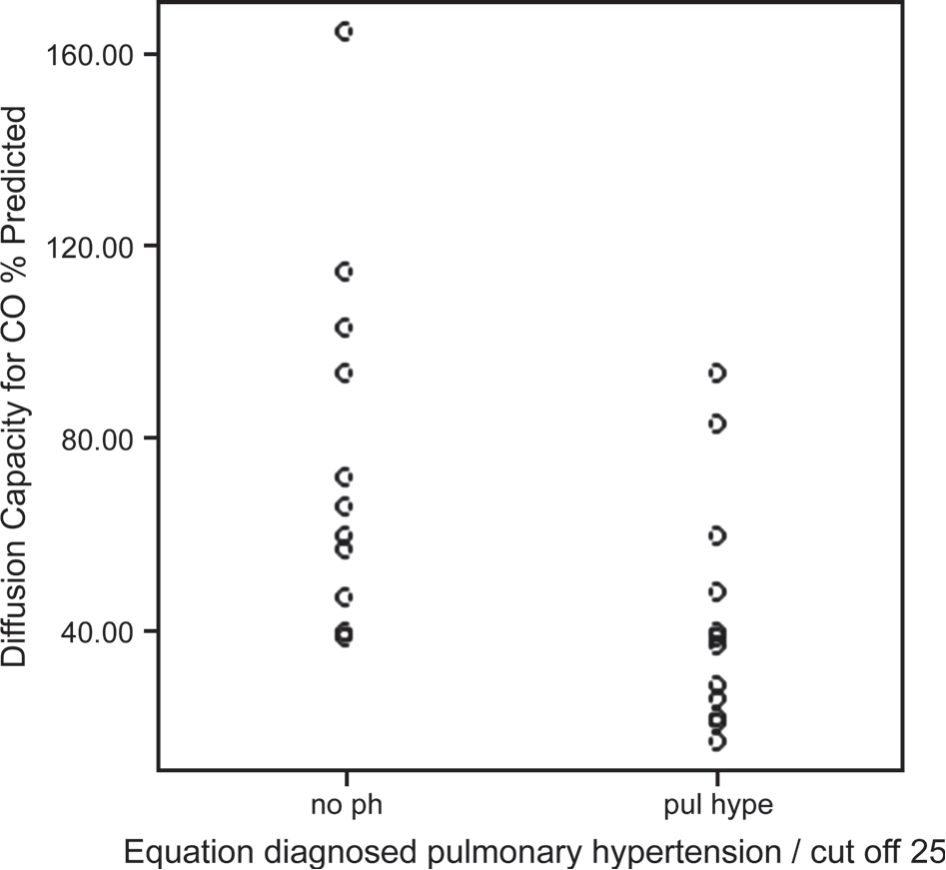
Equation diagnosed pulmonary hypertension in relation to DL_co_% predicted in 37 patients with idiopathic pulmonary fibrosis

**Figure 1b:**
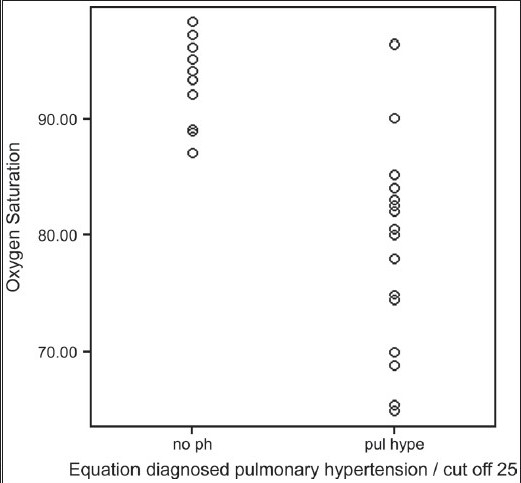
Equation diagnosed pulmonary hypertension in relation to resting room O_2_ saturation in 37 patients with idiopathic pulmonary fibrosis

**Figure 1c:**
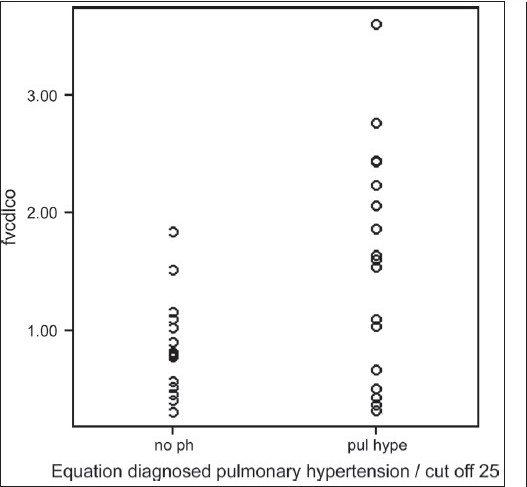
Equation diagnosed pulmonary hypertension in relation to FVC% predicted /DL_co_ % predicted in 37 patients with idiopathic pulmonary fibrosis

**Figure 1d:**
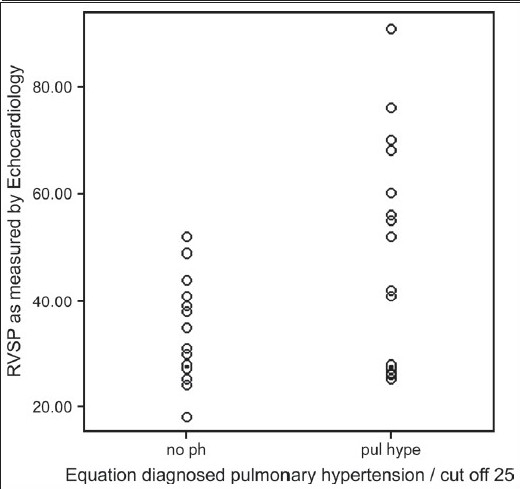
Equation diagnosed pulmonary hypertension in relation to right ventricular systolic pressure in 37 patients with idiopathic pulmonary fibrosis

**Table 3:** Patient characteristics based on formula-predicted presence or absence of pulmonary hypertension using the 25 mmHg cut off point#

Characteristics	No PH N = 18	PH N = 19	*P* value
Age (yr), mean (SD)	44.44 (17.47)	44.79 (16.15)	0.95
Sex (M/F)	15/3	15/4	1
Smoking, n (%)			
Non smokers	15 (83.33)	16 (84.2)	1
Ex-smokers	3 (16.67)	3 (15.8)	1
Chest symptoms, n (%)			
Cough	15 (83.33)	19 (100)	0.11
Grade 1 dyspnea	0 (0)	1 (5.3)	
Grade 2 dyspnea	1 (5.6)	0 (0)	0.5
Grade 3 dyspnea	13 (72.2)	12 (63.2)	
Grade 4 dyspnea	4 (22.2)	6 (31.6)	
Cor-pulmonale, n (%)	11 (61.1)	14 (73.7)	0.5
Central cyanosis, n (%)	14 (77.78)	19 (100)	0.046
Clinical signs of PH, n (%)	4 (22.2)	6 (31.6)	0.71
Electrocardiogram findings, n (%)			
Normal	9 (50)	8 (42.1)	
P-pulmonale	6 (33.3)	11 (57.9)	0.06
Rt ventricular strain	1 (5.6)	2 (10.5)	
Pulmonary function tests, mean (SD)			
FEV_1_, L	1.23 (0.37)	1.00 (0.4)	0.78
FEV_1_, % predicted	52.22 (16.22)	46.16 (14.48)	0.24
FVC, L	1.56 (0.53)	1.37 (0.56)	0.29
FVC, % predicted	54.72 (20.04)	51.58 (17.14)	0.61
FEV_1_/FVC	96.28 (9.44)	87.16 (20.18)	0.09
FVC% pred/DL_CO_% pred	0.86 (0.4)	1.56 (0.91)	0.005
TLC, L	3.33 (0.86)	3.17 (1.39)	0.68
TLC, % predicted	72.83 (21.69)	71.21 (23.00)	0.83
DL_CO_, mL/mm Hg/min	4.95 (2.09)	3.23 (1.7)	0.009
DL_CO_, % predicted	70.89 (32.41)	44.58 (24.7)	0.009
Room air resting arterial blood gases, mean (SD)			
O_2_ saturation	93.44 (3.46)	78.72 (8.38)	0.000
O_2_ tension	67.17 (14.86)	43.6 (10.12)	0.000
CO_2_ tension	38.82 (3.77)	39.46 (10.39)	0.81
Radiographic data, mean (SD)			
CXR findings			
Mean RDPA width	26.72 (10.14)	27.47 (8.8)	0.81
Mean LDPA width	24.89 (7.93)	24.680 (8.22)	0.94
Hilar width in cm	9.67 (2.73)	10.45 (3.33)	0.44
Hilar width > 10.5	6/18	9/19	0.51
Hilar/Thoracic index > 35	12/18	11/19	0.74
Cardiothoracic ratio > 43	11/18	12/19	1.0
CTPA findings			
MPAD in mm	24.78 (8.29)	27.21 (8.76)	0.39
MPAD > 29mm	8/18	12/19	0.33
MPAD > AD > 1	8/18	12/19	0.33
A/B ratio > 1	9/18	12/19	0.52
Cardiac abnormalities	9/18	13/19	0.33
Formula-predicted MPAP, mean (SD)	19.73 (2.82)	48.66 (21.7)	0.000
Echo measured RVSP, mm Hg, mean (SD)	33.99 (8.94)	52.38 (19.68)	0.001

# Data presented as mean (SD) or No/Total. Patients characteristics were available in all of studied 37 patients, PH = pulmonary hypertension, FEV_1_ = forced expiratory volume in first second, FVC = forced vital capacity, TLC = total lung capacity, DL_CO_ = diffusing capacity of the lung for carbon monoxide, RVSP = right ventricular systolic pressure as measured by trans thoracic echocardiography, RDPA = right descending pulmonary artery, LDPA = left descending pulmonary artery, MPAD = main pulmonary artery diameter, AD = aortic diameter, A/B = arterial width/accompanying bronchus diameter

### Correlation between formula-predicted MPAP using cut off point 25 mmHg and putative PH predictors

As shown in Table 4, there were strong and statistically significant correlations in the expected directions between formula-predicted MPAP and TTE-measured RVSP (r = 0.60, *P* = 0.000; Figure 2a), resting room air O_2_ saturation (r = −0.952, *P* = 0.000; Figure 2b) and resting room air O_2_ tension (r = −0.712, *P* = 0.000; Figure 2c).

**Figure 2a:**
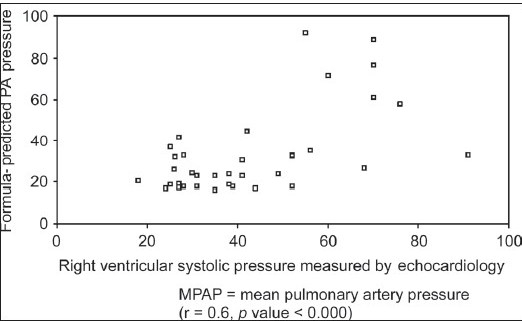
Relationship between formula-predicted mean pulmonary artery pressure and right ventricular systolic pressure as measured by echocardiography in the studied 37 patients with Idiopathic pulmonary fibrosis

**Figure 2b:**
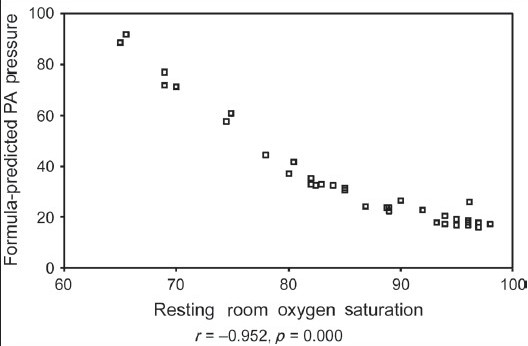
Relation between resting room O_2_ saturation and formula-predicted PA pressure in 37 patients with Idiopathic pulmonary fibrosis

**Figure 2c:**
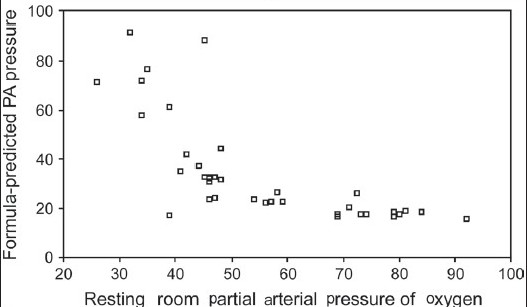
Relation between resting room O_2_ tension and formula-predicted Pressure in the studied 37 patients with Idiopathic pulmonary fibrosis (r = −0.712, *P* = 0.000)

**Table 4:** Pearson's correlation coefficients between formula-predicted pulmonary hypertension using 25 mmHg cut off point and putative predictors of pulmonary hypertension in the studied 37 patients with IPF

Characteristics	*r*	*P* value
Radiological predictors		
Hilar width	0.357*	0.03*
Hilar/thoracic index ≥ 35	0.473**	0.003**
RDPA width on CXR	0.232	0.167
LDPA width on CXR	0.247	0.140
Cardiothoracic ratio	0.019	0.912
MPAD on CT	0.104	0.541
MPAD > 29	0.229	0.173
MPAD/AD > 1	0.229	0.173
A/B >1	0.186	0.270
Pulmonary function tests		
FEV1, L	−0.293	0.078
FEV1, % predicted	−0.276	0.098
FVC, L	−0.066	0.697
FVC, % predicted	0.068	0.688
FEV1/FVC	−0.469**	0.003**
FVC% pred/DL_CO_% pred	0.329*	0.047**
TLC, L	−0.085	0.616
TLC, % predicted	−0.072	0.673
DL_CO_, mL/mm Hg/min	−0.253	0.13
DL_CO_, % predicted	−0.259	0.121
Room air resting arterial blood gases		
O_2_ saturation	−0.952**	0.000**
O_2_ tension	−0.712**	0.000**
CO_2_ tension	0.127	0.453
Echo measured RVSP, mm Hg	0.600**	0.000**

PH = pulmonary hypertension, FEV_1_ = forced expiratory volume in first second, FVC = forced vital capacity, TLC = total lung capacity, DL_CO_ = diffusing capacity of the lung for carbon monoxide, RVSP = right ventricular systolic pressure as measured by trans thoracic echocardiography, RDPA = right descending pulmonary artery, LDPA = left descending pulmonary artery, MPAD = main pulmonary artery diameter, AD = aortic diameter, A/B = arterial/accompanying bronchus diameter;

* Correlation is significant at the 0.05 level (two-tailed);

** Correlation is significant at the 0.01 level (two-tailed)

We observed a modest and significant positive correlation between formula-predicted MPAP and hilar width (r = 0.357, *P* = 0.03), hilar/thoracic index greater than or equal to 35 (r = 0.473, *P* = 0.003), FEV_1_/FVC (r = −0.469, *P* = 0.003), FVC% pred/DL_CO_% pred (r = 329, *P* = 0.047). However, there was no correlation between formula-predicted MPAP and MPAD, MPAD/AD, or the A/B ratio. Similarly, there was no correlation between formula-predicted MPAP and rest of CXR predicted measures for PH including RDPA and LDPA width. Furthermore, using coefficient contingency, we found no correlation between clinical signs of PH and ECG predictors of PH (p-pulmonale, right ventricular hypertrophy) and equation predicted PH (data are not shown).

### Multivariable linear regression of formula-predicted MPAP on other predictors of PH in IPF patients

**Figure 3:**
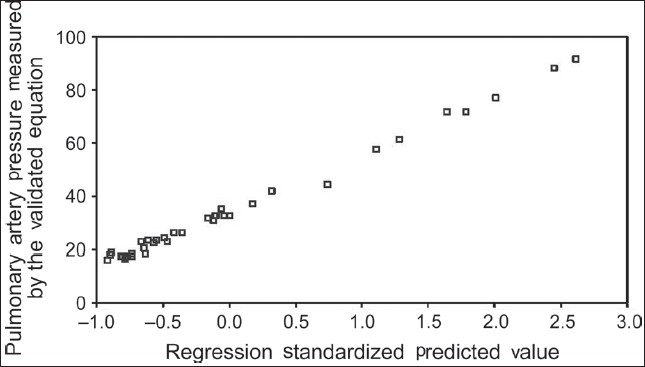
Scatter plot of formula predicted pulmonary artery pressure regression model (Adjusted r^2^=0.969, *P*=0.000)

The 95% CI for the model parameter estimates are listed in Table 5. This model explained 96% of the variance of MPAP (adjusted R2 = 0.969, *P* < 0.000). The model scatter plot of formula-calculated versus model predicted MPAP is shown in Figure 3. Sequential and partial sums of squares associated with the primary predictors demonstrated that SpO_2_ provided the majority of the predictive information.

**Table 5:** Multivariable analysis to assess predictive ability of diagnosing PH in IPF patients using the formula

Variables included	Model coefficients	Sig.	95% confidence interval for B	
			
	Beta		Lower bound	Upper bound
Age	−.004	.975	−.419	.407
Sex	−.213	.053	−23.118	.174
Dyspnea	.023	.852	−8.796	10.359
Clinical signs of PH	−.018	.792	−8.304	6.580
ECG signs of PH	.014	.857	−5.529	6.481
Hilar width	.023	.906	−2.965	3.290
Hilar/thoracic ratio value	.141	.463	−.550	1.089
Hilar/thoracic ratio > 35	−.069	.702	−20.712	14.744
Rt pulmonary artery width	−.070	.397	−.580	.260
Descending LT pulmonary artery width	.057	.540	−.408	.714
Cardio-thoracic ratio > 43	.123	.272	−5.265	15.982
Cardiomegaly on CXR	−.188	.119	−20.794	2.951
Main pulmonary artery width on CT	.232	.398	−.947	2.110
Ratio of pulmonary artery Aorta > 1 on CT	−.762	.069	−67.693	3.297
Diameter of intrapulmonary artery greater than its accompanying bronchus	.636	.016	6.713	47.381
Cardiac abnormalities on CT	0.001	0.981	−5.950	6.078
FEV1 L/min	−0.070	0.784	−35.006	27.482
FEV1 % predicted	0.060	0.787	−0.618	0.785
FVC L/min	0.364	0.185	−8.636	37.019
FVC% predicted	−0.153	0.445	−.696	0.341
FEV1 / FVC ratio	0.054	0.681	−0.315	0.455
Diffusion capacity for carbon monoxide	−0.170	0.190	−4.627	1.107
Total lung capacity % predicted	−0.082	0.354	−0.268	0.110
Residual volume/total lung capacity ratio	−0.008	0.942	−0.145	0.136
Partial arterial pressure of oxygen	0.447	0.015	0.142	0.964
Partial arterial pressure of carbon dioxide	0.092	0.197	−0.167	0.672
Oxygen saturation	−1.343	0.000	−3.553	−2.290
RVSP as measured by echocardiography	0.016	0.836	−0.196	0.235
Diffusion capacity for CO % predicted	0.143	0.358	−0.137	0.333
FVC% pred/ DL_co_% predicted	0.040	0.721	−6.015	8.816

Dependent variable: Equation predicted mean pulmonary artery pressure

## Discussion

This study confirms that the recently validated prediction formula for MPAP using standard lung function measurements can be used as a screening and follow-up tool for PH in IPF patients. In this group of patients, right sided heart catheterization is usually a pre-request for those patients who will undergo lung transplantation; a procedure that is only done in a limited number of centers world wide. In the meantime, the measurements used in this equation for calculation of the MPAP use parameters (SpO_2_, percentage of predicted FVC, and percentage of predicted DL_CO_) that are simple, non-invasive, usually done in the out patient clinics, for assessment and follow-up of those type of patients thereby reducing the need for repeated echocardiography, radiographic assessment of PH including CT angiography or the costly, invasive RHC.

In IPF patients RHC has been the only accepted tool to diagnose PH. Given the invasiveness and cost of this procedure, we aimed to test the reliability of this formula compared to other non invasive approaches usually used to screen for PH in IPF patients to improve patient safety, reduce costs, and enable the appropriate timing of RHC which has limited indications in these type of patients.

Zisman and associates recently demonstrated that the ratio of the forced vital capacity percentage of predicted to diffusing capacity of the lung for carbon monoxide percentage of predicted and room air resting pulse oximetry data can be combined to screen for pulmonary hypertension in patients with idiopathic pulmonary fibrosis,[3] and further did external validation of that formula in another study on a group of IPF patients with advanced disease and recommended others to test the formula on different groups of IPF patients.[7] Our study extends the findings of the previous studies to a more assorted, and clinically relevant, broader population of IPF patients. Moreover, this study addresses important limitations of some of these previous studies in being prospective rather than retrospective.

As Zisman *et al.* advised, clinicians could, depending on circumstances, select either the 21 mm Hg or the 25 mm Hg value as the threshold for confirmatory RHC testing. As we found no statistically significant differences between the mean values and percentages of different patients' characteristics using either cut off point, we preferred to correlate the 25 mmHg cut off point formula-predicted PH with other screening parameters. Based on the prevalence of PH in IPF (approximately 30%),[2,7] we did not want to over diagnose IPF patients who might have PH, because right heart catheterization is not a standard-of-care test in IPF, and usually only one in three RHCs is found positive in this type of patients.[7]

We confirmed that the prediction formula of MPAP of Zisman strongly correlates with TTE measured RVSP, the most commonly used screening tool for PH.[16] We also found a statistically strong correlation with room air PaO_2_ saturation and tension, FVC% pred /DL_CO_% pred, and hilar width on CXR, while chest symptoms, ECG and CTPA signs of PH and rest of radiological indices poorly correlated with the same formula.

As seen in this study, the clinical cardinal symptom of PH is dyspnea.[15,16] IPF patients enrolled in this study had different range of their grade of dyspnea. Despite that most of them had grade III (67.6%), 27% were in grade IV, and only 5.14% of patients were still in grade I and II. However, IPF patients are usually short of breath regardless they developed PH or not, yet, the clinical suspicion of PH in those patients usually arises when the level of dyspnea is unexplained by the level of severity of the underlying lung disease.[16]

Other methods to predict presence PH in IPF are not as reliable. Electrocardiography lacks sufficient diagnostic accuracy to serve as a screening tool for the detection of pulmonary arterial hypertension.[15] Right ventricular hypertrophy on ECG is present in 87% and right axis deviation in 79% of patients.[27] ECG has inadequate sensitivity (55%) and specificity (70%).[28] A normal ECG does not exclude the presence of severe pulmonary hypertension.[15] This is in agreement with our results where 42.1% with formula-predicted PH showed normal ECG trace, compared to 57.9% of patient who had either P-pulmonale with or without ECG signs of right ventricular strain in their ECG trace. In concordance with our results the chest radiograph was abnormal in 90% of pulmonary arterial hypertension patients at the time of diagnosis. In this study hilar width and hilar-thoracic ratio with a value greater than 0.44 strongly correlated with formula-predicted PH.[15,27] However, a normal chest radiograph does not exclude mild pulmonary hypertension including left-heart disease or pulmonary veno-occlusive disease.[15]

Although Doppler echocardiography (DE) is recommended as a screening tool for the diagnosis of PH,[5,10,15,16] its accuracy in estimating pulmonary artery systolic pressure in PH patients has been questioned. It had been shown that echocardiography-estimated right ventricular systolic pressure predicted PH poorly in IPF patients (76% sensitivity, 38% specificity, 56% PPV, and 60% NPV).[3] In a previous study,[17] echocardiography predicted PH in patients with various interstitial lung disease (ILD) with 85% sensitivity, 17% specificity, 60% PPV, and 44% NPV. DE can frequently overestimate and underestimate pulmonary artery pressure in PH patients. This error is in part explained by inaccuracies of right atrial pressure estimation and poor Doppler imaging of the transtricuspid regurgitant jet. Particular caution should be exercised in assessing PA pressure by DE when the TR jet quality is low, as serious pressure underestimations can occur, leading to missed or delayed diagnosis of a disease with high morbidity and mortality. Also, the estimation of cardiac out put by DE does not appear reliable. Fisher *et al*.[29] said that DE may not be very useful when used serially in assessing changes in pulmonary artery pressure in response to therapy, due to significant individual over and underestimation of pressure which underscores the importance of taking other echo-derived metrics (i.e., measures of RV size and function) into consideration as well.[29]

On CTPA, pulmonary artery dilatation occurs in the absence of PH in patients with pulmonary fibrosis and is therefore an unreliable sign of PH in these patients.[24] This has been noticed in our group of IPF patients, many of them had advanced disease when enrolled, where MPAD greater than or equal to 29 mm on CTPA poorly correlated with the formula-predicted PH (r = 0.229, *P* = 0.173). However, previous studies of the association between pulmonary artery size and pulmonary artery pressure have been inconsistent, with some investigators[30–32] finding correlations in the expected direction, and others[33–35] reporting no correlation. Our results support the previous studies[33–35] that have found no correlation between pulmonary arterial diameter and pulmonary artery pressure. It should be emphasized that our study population consisted of a group of IPF patients, whereas other investigators[36,37] have focused on a wide spectrum of cardiopulmonary diseases, with a large proportion of patients with pulmonary vascular disease (PVD) such as idiopathic pulmonary arterial hypertension or chronic thromboembolism.[38,39] In previous studies[40,41] that have found associations between pulmonary artery size and PH, the PH cases were predominantly composed of patients with PVD with greater pulmonary artery pressure than our IPF patients with PH. Our study is consistent with these findings and together they suggest that PH due to IPF may not increase MPAD. It is also conceivable that the restrictive lung physiology in IPF may result in a traction effect on the mediastinal vascular structures distending the pulmonary artery independent of the underlying pulmonary artery pressure; this effect may dampen the influence of the pulmonary artery pressure on the MPAD in IPF patients.

Elevated serum level of brain natriuretic peptide is associated with moderate-to-severe PH (MPAP 35 mm Hg) with PPV of 73% and NPV of 92%.[42] However, the sensitivity of brain natriuretic peptide to detect mild-to-moderate PH (MPAP of 26 to 34 mm Hg) is unknown, and this method has not been validated.

Recent studies suggest that DL_co_ less than 40% pred and need for oxygen supplementation are predictive of PH in patients with IPF and in sarcoidosis.[2,5,43–45] In a study by Lettieri *et al*.[2] PH was present in 31.6% of patients. However, the predicted prevalence of PH was 15.2%, suggesting that a prediction based on DL_CO_ alone and the need for supplemental oxygen would not identify 50% of the PH cases. By employing the extent of desaturation rather than the need for oxygen and the FVC/DL_CO_ ratio in place of DL_CO_, the prediction method described by Zisman *et al*.[7] increased both sensitivity and NPV.

### Limitations of study

There are some limitations of this study. First, the sample size is not large; as only IPF patients who had all data collected at the same time of enrollment were included in analysis. Patients were not subjected to RHC; hence, we could not correlate the formula-estimated MPAP with actual RHC measured values. Unfortunately, because of the cost of RHC, we do not usually catheterize IPF patients as it is usually a pre-request for lung transplantation.[7,15] A low formula-predicted MPAP during a single evaluation does not rule out the possibility of PH developing in the future. However, the formula-predicted MPAP can be followed on a serial basis because it is computed using clinical variables that are routinely measured. Most of the patients in this series were in relatively advanced stage of their illness. However, the study included limited number of IPF patients who were in earlier stages. We believe that testing the formula on larger number of IPF patients in their early stage is warranted to prove if it is reproducible in such group of patients with limited impairments of their ABGs and DL_co_ levels, still we believe it can work as a simple routine follow-up tool for them.

### Strengths

First, the study establishes the empirical validity of a new, easy-to-use, clinical screening method for PH in IPF patients. Second, this study shows that the formula can be applied in IPF populations at any medical center, that is, the method is transportable. Third, the cut off formula-predicted of 25 mm Hg is as equal as TTE in predicting PH in those patients and this would prevent unnecessary repetition and cost of other screening tools in this group of patients. Also, our study, included IPF patients at relatively earlier stages of their disease not only those who were candidates for lung transplantation. Moreover, it limits the number of patients who need to undergo confirmatory invasive and expensive RHC.

## Conclusion

This formula-predicted MPAP using standard lung function measurements is a simple non invasive screening and follow-up tool for PH in IPF patients.
